# Simultaneous Proton Transfer Reaction-Mass Spectrometry and electronic nose study of the volatile compounds released by *Plasmodium falciparum* infected red blood cells *in vitro*

**DOI:** 10.1038/s41598-019-48732-x

**Published:** 2019-08-26

**Authors:** Rosamaria Capuano, Iuliia Khomenko, Felicia Grasso, Valeria Messina, Anna Olivieri, Luca Cappellin, Roberto Paolesse, Alexandro Catini, Marta Ponzi, Franco Biasioli, Corrado Di Natale

**Affiliations:** 1 Department of Electronic Engineering, University of Rome Tor Vergata, Via del Politecnico 1, 00133 Roma, Italy; 2 Department Food Quality and Nutrition, Fondazione E. Mach., Via E. Mach 1, 38010S Michele all’Adige, TN Italy; 3 Department of Infectious Diseases, Istituto Superiore di Sanità, Viale Regina Elena 299, 00161 Roma, Italy; 4 Department of Chemical Sciences, University of Padua, Via F. Marzolo 1, 35131 Padova, Italy; 5 Department of Chemical Science and Technology, University of Rome Tor Vergata, Via della Ricerca Scientifica, 00133 Rome, Italy

**Keywords:** Analytical chemistry, Parasitology

## Abstract

The discovery that Volatile Organic Compounds (VOCs) can be biomarkers for several diseases has led to the conception of their possible application as diagnostic tools. In this study, we aimed at defining of diagnostic signatures for the presence of malaria transmissible stages in infected individuals. To do this, we compared VOCs released by asexual and sexual stage cultures of *Plasmodium falciparum*, the deadliest species of malaria, with those emitted by uninfected red blood cells (RBCs). VOC analysis was carried out with an innovative set-up, where each sample was simultaneously analysed by proton transfer reaction time of flight mass spectrometry (PTR-ToF-MS) and an electronic nose. PTR-Tof-MS results show that sexual stages are characterized by a larger emission of hexanal, compared with uninfected or asexual stage-infected RBCs, which makes them clearly identifiable. PTR-Tof-MS analysis also detected differences in VOC composition between asexual stages and uninfected RBCs. These results have been substantially replicated by the electronic nose analysis and may open the possibility to develop sensitive and easy-to-use devices able to detect sexual parasite stages in infected individuals. This study also demonstrates that the combination of mass spectrometry with electronic noses is a useful tool to identify markers of diseases and to support the development of optimized sensors.

## Introduction

Malaria is a serious worldwide health problem, with a total of 216 million cases in 2016 involving 445,000 deaths, most of them in Africa^1^. The majority of malaria deaths are caused by *Plasmodium falciparum*, the most virulent of the *Plasmodium* species. *P. falciparum* has a complex life cycle that involves two types of host: humans and female *Anopheles* mosquitoes. In humans, an asymptomatic multiplication in liver cells is followed by parasite release into the bloodstream and erythrocyte invasion by specialized invasive forms called merozoites. Once inside erythrocytes, parasites develop into trophozoites that replicate their DNA by schizogony. Mature schizonts rupture releasing a new generation of invasive forms that undergo a new asexual cycle. A subset of this population instead develops into sexual stages – the gametocytes – responsible for malaria transmission. Gametocytes develop through five distinct stages (I-V). Immature sexual stages (I-IV) are mainly sequestered in the bone marrow, while mature stage V gametocytes are released into the circulation, becoming accessible to mosquitoes during the blood meal.

In the last 15 years, malaria incidence and mortality rate have considerably decreased thanks to vector control and chemoprevention. Moreover, the increasing availability of rapid diagnostic tests (RDTs) has contributed to improving diagnostic accuracy and, consequently, to the administration of appropriate treatments^2^. Among the licensed anti-malarial drugs, only primaquine is effective against late stage *P. falciparum* gametocytes but its use is limited as it may cause haemolytic anaemia in G6PD-deficient individuals^3^. Consequently, treatment with current antimalarial drugs often results in asymptomatic carriers who remain infectious for weeks after the clearance of asexual parasites.

Achieving malaria eradication requires targeting the human reservoir of sexual parasite stages, responsible for transmission to the mosquito vector. Parasite carriers often harbour sexual stages at submicroscopic levels, only be detectable by molecular methods. In this context, identification and monitoring of gametocyte reservoirs is a fundamental aspect. To this aim, the development of sensitive, non-invasive, easy-to-use and low-cost devices to detect parasite sexual stages in infected individuals is strategic.

Several studies in human malaria showed that *Plasmodium*-infected individuals are more attractive to mosquitoes than non-infected hosts, particularly when high levels of gametocytes are present^4,5^. However, it remains unclear whether the increased human attractiveness to mosquitoes is due to infection in general.

Emission of malaria-specific volatile organic compounds (VOCs) has been shown in *Plasmodium* infected mice^6,7^ and in skin^8^ and breath^9^ samples from malaria-infected humans. The search of specific VOCs released by *P. falciparum* in *in vitro* cultures produced conflicting results; even if these studies shared a similar experimental approach based on the collection of VOCs on solid-phase microextraction (SPME) followed by gas chromatography-mass spectrometry (GC-MS) analysis. Wong *et al*. did not identify any malaria related VOCs^10^, Kelly *et al*. evidenced an over production of terpenes^11^ and finally Correa at al. evidenced a clear correlation of hexanal with malaria infection^12^. None of these studies considered a possible specificity of VOCs emitted from different stages of *Plasmodium* parasites.

With the aim of investigating whether different parasite stages emit distinct stage-specific compounds, we analysed the VOCs released by asynchronous asexual stages, and gametocytes at different stages of maturation.

The analysis had an innovative setup where a Proton Transfer Reaction Mass Spectrometer (PTR-MS) was hyphenated with a gas sensor array, enabling the simultaneous measurement of the same sample with both techniques. PTR-MS enables real-time detection of VOCs with low detection limits. This feature has been frequently exploited for medical studies to characterize VOCs released both *in-vivo* and *in-vitro*^13^. In this study, we used a PTR-MS based on a time-of-flight mass analyser (PTR-ToF-MS). PTR-ToF-MS ensures a higher throughput and a better mass resolution compared to the more traditional quadrupole mass analyser^14^.

Gas sensor arrays (also known as electronic noses) are a valid alternative to costly and bulky instruments and are being developed as ‘field’ tools^15^. They have been often applied to detect several pathologies in *in-vivo* and *in-vitro* studies^16^. Recently, this technology was shown to be sufficiently sensitive and selective to detect the progression of malaria infection in a murine model^7^.

In this study, the real-time detection of PTR-ToF-MS was combined with gas sensors in order to simultaneously measure the same sample, thus avoiding possible artefacts linked to multiple independent measurements. The combined use of different instruments is used in analytical chemistry to compensate the limitations of the individual techniques but, in this case, it has an additional goal. Electronic noses provide scarce information about the nature of the detected compounds, therefore the information retrieved by PTR-ToF-MS helps to elucidate the working mechanism of the gas sensors and may eventually be used to support the development and optimisation of application-oriented sensor arrays.

The results of PTR-ToF-MS and gas sensors showed significant differences in VOCs emissions from gametocytes-infected RBCs respect to those released by RBCs infected with asexual stages. In particular, gametocyte infection is accompanied by a strong emission of hexanal. These results showed that different stages of the parasite emitted discriminative VOCs with stage-specific distinctive features. Furthermore they indicated that instruments for the specific identification of malaria transmissible stages can be devised.

## Results

### Experimental design

**Figure 1:**
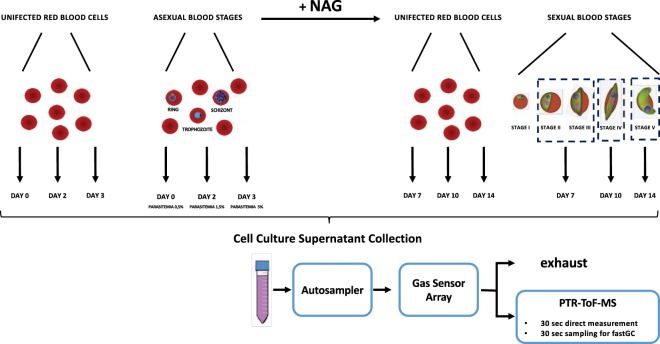
Experimental set-up: RBCs infected with *P. falciparum* asexual stages were cultured for four days in two biological replicates. Supernatants were sampled at day 0 (0.5% parasitemia in both replicates), day 2 (parasitemia 1.3% and 1.6%) and day 3 (parasitemia 4.2% and 5.9%). At day 4, NAG was added to selectively eliminate the asexual forms. For sake of comparison, NAG was also added to the intact RBC. Gametocyte culture supernatants were sampled at day 7, gametocytes stage II-III (gametocitemia 1.6% and 2.2%); at day 10, gametocytes stage IV and at day 14, gametocyte stage V. Uninfected RBC cultures used as a control were sampled in parallel. Collected supernatants were measured in sequence with electronic nose and PTR-MS.

In order to investigate changes in VOCs emitted by *P. falciparum* asexual and sexual stages *in vitro*, asexual forms were induced to produce fully mature gametocytes in 10–14 days by overgrowth in blood cultures. The gametocytes were then selected by supplementation with N-acetylglucosamine (NAG) that is selectively toxic for asexual trophozoites and schizonts, while not affecting gametocytes^17^. Supernatants from cultures of asynchronous asexual blood stages were collected at three time points (days 0, 2 and 3), with a parasitemia ranging from 0.5% to 5%. The supernatant from sexual stage cultures was also collected at three time points (days 7, 10 and 14), corresponding to stage II-III gametocytes, stage IV gametocytes and mature gametocytes. As a control, we also collected supernatants from uninfected erythrocytes maintained in the same culture conditions. After centrifugation to remove healthy and parasitized erythrocytes, equal volumes of supernatants were divided in three aliquots and placed in sealed vials for headspace volatile compound analysis. Measurements were performed on two independent biological replicates. The experimental setup is shown in Fig. 1.

### PTR-ToF-MS analysis

In PTR-ToF-MS spectra, 405 masses were identified in the range between m/z = 21.02 and m/z = 205.19. Most of these compounds are found at concentrations  smaller than 1 ppb.

**Figure 2:**
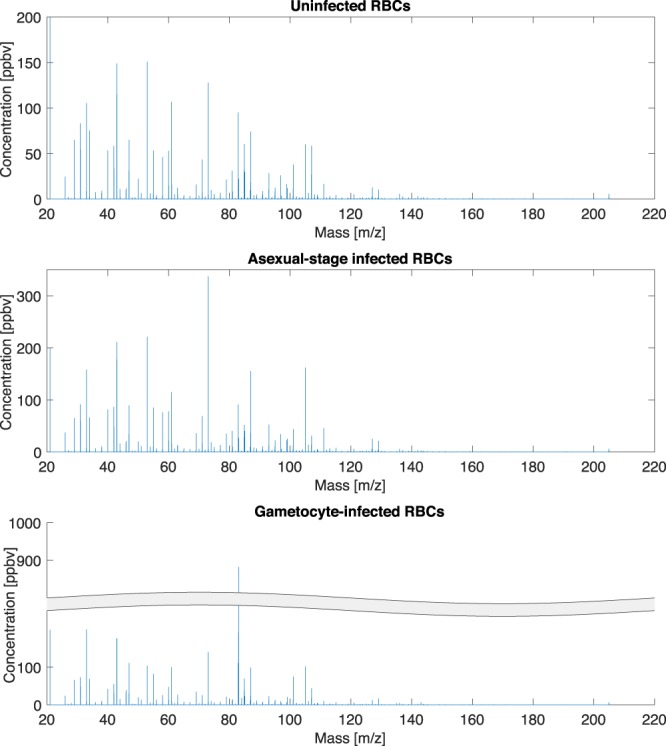
Example of PTR-ToF-MS spectra for each group. The y axis of gametocyte-infected RBC sample is split in order to accommodate the high peak at m/z = 83.086. Figures represents the raw data where the abundance at m/z = 83.086 is overestimated.

A visual inspection of the spectra showed a certain homogeneity among the samples. Figure 2 shows representative PTR-ToF-MS spectra for each of the three groups: uninfected RBCs and RBCs infected with either asexual or sexual blood stages. A comparison of these spectra shows only subtle differences between groups, except for the abundance at m/z= 83.086. The concentration of this compound appears to be one order of magnitude larger in gametocytes respect to the other groups. This mass corresponds to the formula C_6_H_11_^+^ identified as hexanal (dehydratation fragment). The identification of the peak at m/z = 83.086 as a fragment of hexanal is supported by: the exact mass, the presence in the spectra of the isotopologue at m/z 84.089, and the correlation with the parent ion at m/z 101. Furthermore, the identity of this peak as hexanal was confirmed by fast GC analysis of the gametocyte samples. The highest concentration of hexanal is found in gametocytes at stages IV and V.

**Figure 3:**
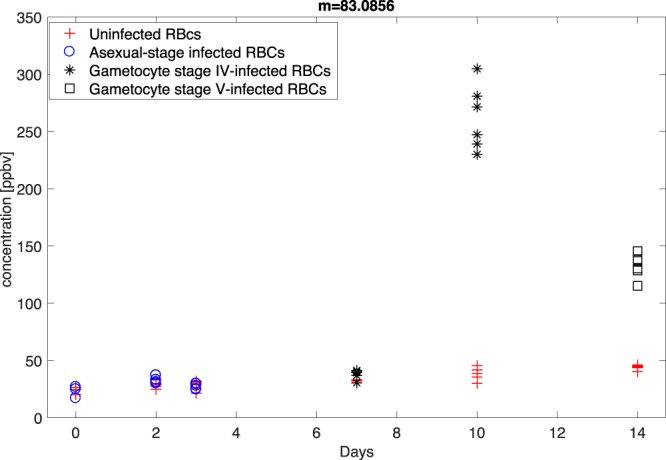
Concentration of m/z = 83.0856 in all samples. This mass is identified as hexanal (dehydratation fragment). The concentrations released by RBCs infected with gametocytes at stages IV and V are evaluated from the concentration of the isotopologue at m/z = 84.089.

The amount of hexanal in these samples is high enough to induce saturation at m/z = 83.086. In this case the concentration of the peak m/z = 83.086 was estimated from the concentration of the isotopologue at m/z = 84.089 assuming the expected isotopic abundance of 6.88% that was experimentally confirmed for lower concentrations (see Fig. S1 in Supplementary Information File). During fastGC measurements the correct abundance ratio between m/z = 83.086 and m/z = 84.089 was observed at the corresponding chromatographic peak also for these samples. Figure 3 shows the amount of hexanal (m/z = 83.086) in all samples.

The total concentration of hexanal, as the sum of the two masses, reached about 0.5 ppm in stage IV gametocytes at day 10. In stage V gametocytes at day 14, the concentration of hexanal dropped to 20% of that observed in stage IV gametocytes but still well above the concentration found in both uninfected RBCs and RBCs infected with the asexual stages.

The peculiarly high concentration of hexanal made gametocyte-infected RBCs readily identifiable compared with uninfected or asexual stage-infected RBCs. However, we were also interested to expand the analysis of gametocyte-specific VOCs and possibly identify features that distinguish uninfected from asexual -infected RBCs. For this scope, the statistical distribution of the concentration of each mass peak between different groups was evaluated with a non-parametric Kruskal-Wallis rank sum test. The results (see Fig. S2 in Supplementary Information File), expressed as the null hypothesis probability (p-value), show that in gametocyte samples, the concentration of 54 peaks is statistically different (p < 0.01). On the other hand, only 9 peaks allowed the separation of uninfected and asexual -infected RBCs (p < 0.01).

In order to investigate the collective properties of the detected peaks and the composition of the volatile compound patterns, a principal component analysis (PCA) of PTR-ToF-MS spectra was used without normalization to emphasise the contribution of the most abundant compounds. This choice may hide other possibly meaningful compounds occurring at low concentrations but, conversely, underlined those compounds which were more easily detectable by gas sensors.

**Figure 4:**
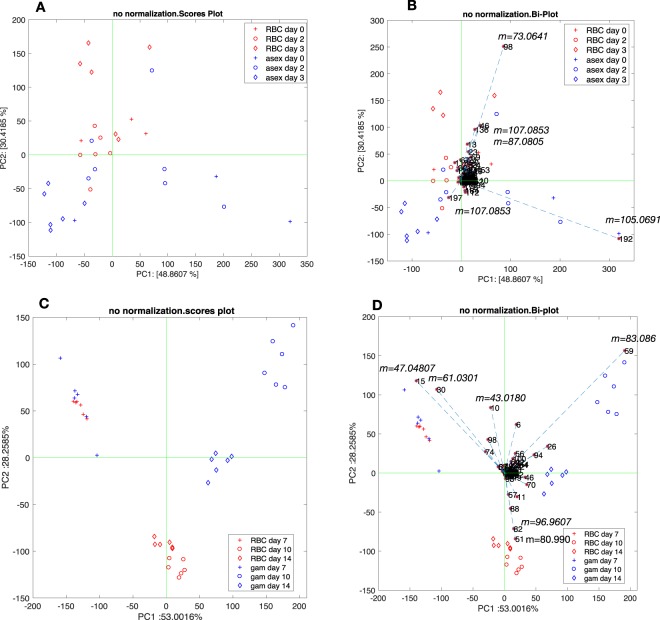
Results of PCA of PTR-MS spectra. (**A,B**) Shows scores and biplot of the PCA data on uninfected and asexual stage-infected RBCs. All variables in the biplots are labelled with the order number in the spectra. More significant variables are labelled with the corresponding mass. Uninfected RBCs (RBC); asexual blood stages (asex); gametocytes (gam). (**C,D**) Show the scores and the biplot of the PCA data on uninfected and gametocyte-infected RBCs collected at day 7, immediately after the addition of NAG with gametocytes at stage III, and at days 10 and 14 with gametocytes at stage IV and V respectively. Data are labelled as: Uninfected RBCs (RBC); asexual blood stages (asex); gametocytes (gam).

Figure 4A,B show the results of the PCA of the PTR-ToF-MS spectra in the first part of the experiment (days 0–3) when uninfected and asexual stage-infected RBCs were measured.

The representation includes the two first principal components that account for about 79% of the total variance of the data (see Fig. S3 in Supplementary Information File). The two first principal components provide a better separation among the groups as shown by the non-parametric Kruskal-Wallis rank sum test (see Fig. S4 in Supplementary Information File). The scores plot in Fig. 4A shows a partial separation between the two groups, with discrimination between uninfected and asexual-infected samples occurring along the second principal component axis, explaining 30% of total variance (Fig. 4A). Figure 4B highlights those compounds that contribute the most to the data representation in the first two principal components. The separation of asexual stage-infected RBC samples is mostly due to peaks at m/z 105.0691 and 107.0853. They are putatively identified as styrene and ethylbenzene respectively.

Figure 4C,D show the scores plots and the biplots of uninfected- and gametocyte-infected RBCs collected at days 7, 10 and 14, which corresponded respectively to stage III, IV and V of gametocyte maturation. The first two principal components explain more than 80% of variance (see Fig. S5 Supplementary Information File).

The non-parametric Kruskal-Wallis rank sum test applied to the principal components shows that the first two principal components are more discriminant respect to the two groups (see Fig. S6 in Supplementary Information File).

Due to its high abundance, hexanal (m/z = 83.086) played a major role in separating the stages IV and V gametocytes from the other groups. Hexanal dominated the first principal component that explained >86% of total variance.

Uninfected and infected RBC samples at day 7 were not distinguishable from each other but separate from other samples; Fig. 4D indicates the peaks most responsible for this separation. Among them, the peak at m = 47.04807 and m = 61.0301 are putatively identified as ethanol and acetic acid respectively. These compounds are likely related to the metabolic products of supplemented NAG. Acetic acid, for instance, has been found associated with glucosamine and N-acetyl glucosamine in fungal cell walls^18^. Another peak aligned towards the samples at day 7 corresponds to m = 43.0180. This is an unspecific fragment whose origin may be attributed to several compounds.

### Gas sensor array

**Figure 5:**
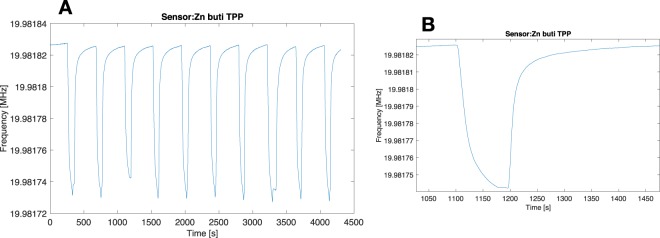
(**A**) Sequence of signal of one of the sensors, exposed to a series of samples. (**B**) Details of one of the measurements.

Gas sensors were exposed to the same samples of the PTR-ToF-MS. Figure 5 shows a portion of signals of one of the sensors of the array. The exposure time and the sample flow were enough to get a stable and reproducible sensor signal. It is interesting to remark that after exposure to each sample, the sensor signal quickly recovers its initial conditions. The shift of frequency, calculated as the difference of signal at the end of the exposure and immediately before the sample, was considered as the sensor response and it was used in the following analysis.

The aptitude of each sensor to discriminate between the different groups of samples was evaluated with a non-parametric Kruskal-Wallis rank sum test.

**Figure 6:**
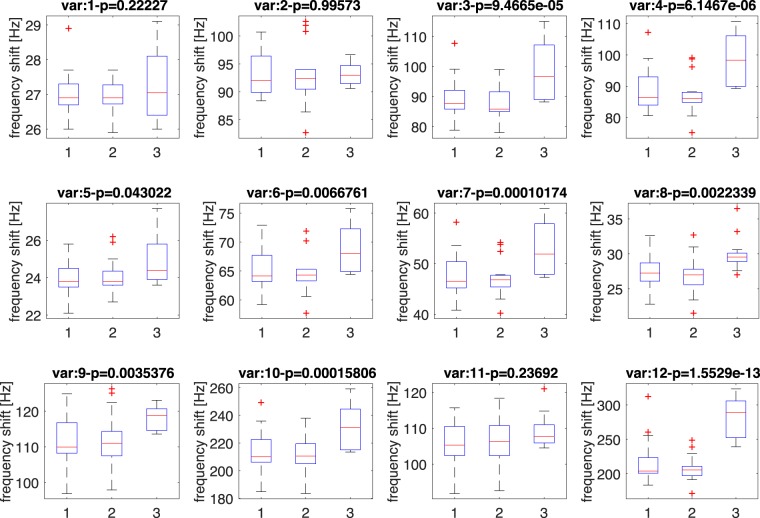
Variance test of sensor data. Each box-plot illustrates the statistical distribution of the corresponding sensor. The header of each box-plot reports the p-value related to at least the separation of one of the three groups respect to the others. Classes are labeled as 1: RBCs, 2: asexual parasites, 3: gametocytes.

Variance tests were performed for each sensor, the statistical distribution of the sensor responses to the different classes are shown in Fig. 6. Sensor responses are in good agreement with the PTR-ToF-MS data. In particular, most the sensors were sensitive to the excess of compounds released by gametocytes, while the differences between asexual stage-infected red blood cells and uninfected RBCs was not evident. On the basis of the variance analysis, a reduced array was selected omitting those sensors that showed a p-value > 0.01. On this basis, sensors 1, 2, 5, and 11 were excluded from the following analysis.

**Figure 7:**
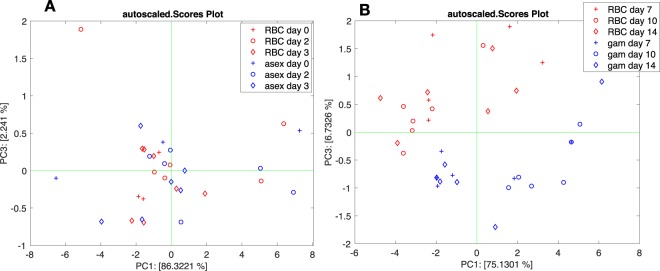
Scores plot of the PCA calculated with asexual stage-infected RBCs (**A**) and gametocyte-infected RBCs (**B**).

PCA was applied to the subset of sensors, and it was calculated on standardized data (zero mean and unitary variance). Scaling was applied to eliminate the influence of any variation in sensors signal magnitude, likely due to uneven sensors preparation. PCA was calculated separately for the two experimental parts, before and after the addition of NAG. Variance testing was applied to the principal components to select those providing the best representation of the data. The scores plot of the two PCA models are shown in Fig. 7.

Electronic nose data do not show any separation between uninfected and asexual stage-infected RBCs. The distribution of the principal components in the two groups are almost overlapped (Fig. S7 in the Supplementary Information File) with a slight separation achieved by PC1 and PC3. The plot of the corresponding scores (Fig. 7A) showed a substantial overlap among the groups.

A different pattern was observed after the addition of NAG. The statistical distribution of the principal components respect to non-infected RBCs and gametocytes is shown in Fig. S8 in the Supplementary Information File. Variance analysis shows that PC1 and PC3 achieved best separated the two groups (Fig. 7B) with 81% of the total variance explained. The result is qualitatively similar to that obtained by PTR-ToF-MS (Fig. 4C) with the noteworthy difference that the data in each group were not structured and in particular there was no separation within the gametocyte group associated with different maturity stages. Furthermore, we did not observe the peculiar separation of samples collected at day 7 as in the case of PTR-ToF-MS. This suggested a lack of selectivity towards the NAG signaling compounds. However, the separation between gametocytes and uninfected RBCs was complete and, due to the missing influence of the addition of NAG, gametocytes were also identifiable at day 7.

## Discussion

In this study we analysed the VOC patterns from supernatants of non-infected RBCs and RBCs infected *in vitro* with the human malaria parasite *P. falciparum* by both Proton Transfer Reaction based on a time-of-flight Mass Spectrometer (PTR-ToF-MS) and Gas Sensor Array. The main objective of this study was to identify features in VOC emission distinctive of either asexual or sexual blood stages of the parasite.

The analysis of VOCs from cell culture supernatant is an established methodology. However, it is worth considering that the composition of the supernatant headspace may differ from that actually released by cells. In particular, molecules characterized by a large volatility are expected to be lost during supernatant separation. Thus, molecules with higher solubility and higher partition coefficient at air/liquid interface are preferentially retained and a bias in the pattern of volatile compounds may be introduced.

On the other hand, in *in-vivo* conditions, metabolites present in the blood are either exchanged at the air/blood interface in the lung or, in alternative, released in air via subdermal capillaries.

The different method of sample collection, together with the fact that VOCs have different partition coefficient in different media, may contribute to explain the discrepancy between our results and the two previously published *in vivo* studies that investigated VOCs in breath^9^ and in skin^8^. Eventually, the partition coefficients of VOCs in different contexts deserve to be carefully scrutinized^19^.

PTR-ToF-MS offers several advantages compared with other analytical instrumentation, among them on-line operation, high-sensitivity and limited fragmentation so that most molecules give a single peak. However, as a direct injection mass spectrometric method, the identification of compounds is only carried out by the exact mass which provides only the sum formula. Thus, there is a non-negligible uncertainty in the identification of isomeric compounds.

PTR-ToF-MS results show that enough molecules are retained in the supernatant, and that the concentration of many of them was altered when RBCs were infected by *Plasmodium*. Non-parametric Kruskal-Wallis rank sum test showed that 54 PTR-ToF-MS peaks are statistically different (p < 0.01) between gametocyte-infected and uninfected RBCs, while only 9 PTR-ToF-MS peaks are significantly different between uninfected and asexual stage-infected RBCs. This difference clearly indicated the dramatic metabolic changes that occurred between asexual and sexual *Plasmodium* blood stages, such as the exceptionally high emission of hexanal in stage IV gametocytes. Hexanal is a common molecule released by living beings. It is ubiquitous in many body compartments^20^ and it has been found altered in response to differentiation of stem cells^21^.

A similar study found anomalous concentrations of hexanal in the supernatant of *Plasmodium*-infected RBCs compared to non-infected cells^12^. Respect to this previous finding, we were able to specifically link high hexanal production to mature gametocytes. Interestingly, hexanal has also been also found among altered VOCs in the skin of malaria-affected individuals^8^.

Finally, it is important to remark that hexanal is a common attractant for many insects, including the malaria vector *Anopheles gambiae*^22^. The combination of all these observations suggests that hexanal, excreted from gametocytes in blood and possibly liberated in air may represent a key molecule in parasite transmission from man to mosquito.

An effective control towards the final goal of malaria eradication, has to face the major challenge of the highly efficient spread of the disease. Since sexual stages are rarely detected by microscopy, it was long assumed that only a small fraction of malaria infected individuals were capable of infecting mosquitoes. It has now been demonstrated that gametocytes are instead highly prevalent in infected individuals^23^. Moreover, mosquitoes can become infected also by biting asymptomatic individuals with very low gametocyte densities, often only detectable by molecular assays^24^.

This paper provides evidence that cultures of transmissible stages of the human malaria parasite, *P. falciparum*, release specific VOCs that allow for their identification and suggests that devices able to monitor gametocyte carriers may be conceived to support malaria control. Extension to the analysis of skin and breath volatile compounds in model animals and humans is envisaged.

Multivariate analysis of PTR-ToF-MS spectra showed that also asexual stage-infected RBCs can be distinguished from non-infected RBCs. While the identification of gametocytes is largely driven by hexanal, in the case of asexual stages, ethylbenzene and styrene, putatively identified by mass comparison, are among the molecules whose concentration is most altered.

Although their biological origin is not totally clear^25^, these molecules have been found in the headspace of cultured cells^21^ and in breath of lung cancer-affected individuals. It is interesting to note that ethylbenzene was previously identified among the VOCs released by skin and discriminative for malaria infection^8^.

The multivariate analysis of PTR-ToF-MS data was performed without scaling the concentration of compounds. Consequently, the more discriminating compounds were also those occurring at higher concentrations and the ones that had the largest probability to reach the outer environment once excreted.

PTR-ToF-MS analysis was complemented by the electronic nose. It is worth remarking that this is the first time that an electronic nose is hyphenated with a PTR-ToF-MS. The electronic nose used in this study was based on sensors shown to be sufficiently sensitive and selective to capture changes in VOCs patterns associated with several pathologies such as lung cancer^26^, tuberculosis^27^, and *in vitro* stem cells differentiation^21^.

As expected, the electronic nose data are less detailed compared to PTR-ToF-MS, but gametocyte-infected RBCs are clearly distinguished from uninfected RBCs, while no differences were detected between uninfected and asexual-infected RBCs. It is conceivable that host-parasite interaction may contribute to the emission of discriminative VOCs, since the same electronic nose was also applied to study changes of volatile compounds in a murine model of malaria^7^.

In spite of the easiness of the analysis and the relative low-cost of the device, electronic noses have many drawbacks such as the lack of information about VOCs detected and discrimination among samples. For this reason, electronic noses are typically complemented by analytical instruments and GC-MS is the standard choice for these applications. However, GC-MS does not ensure an in-line measurement, furthermore, analytical studies and electronic nose analysis are made on split samples measured at different times. Headspace composition is rather fragile and it tends to change due to a number of factors, e.g. temperature, chemisorption or physisorption. All these drawbacks are clearly reduced when the same samples are simultaneously measured.

The coupling of direct injection mass-spectrometric methods with gas sensors is a promising way for an optimal developing and testing of tailored gas sensors.

## Materials and Methods

### Parasites cultures

Asynchronous *Plasmodium falciparum* 3D7 parasites were maintained in RPMI 1640 medium containing 10% heat-inactivated human serum, hepes (5.94 g/l), hypoxanthine (0.05 g/l), sodium bicarbonate (2 g/l) and gentamycin (20 mg/l) in the presence of human group O+ erythrocytes at 5% haematocrit. Cultures were incubated at 37 °C in a tri-gas mix of 2% O_2_, 5% CO_2_, and 93% N_2_.

Asynchronous *Plasmodium falciparum* 3D7 parasites were maintained in RPMI 1640 medium containing 10% heat inactivated human serum, hepes (5.94 g/l), hypoxanthine (0.05 g/l), sodium bicarbonate (2 g/l) and gentamycin (20 mg/l) in the presence of human group O+ erythrocytes at 5% haematocrit. The Transfusion Unit at the University La Sapienza in Rome provided blood from healthy donors after obtaining informed consent.

To induce gametocyte formation, asexual parasites were subjected to stress conditions (high parasitaemia). Cultures were then supplemented with 50 mM N-acetyl glucosamine (NAG), Sigma-Aldrich, to eliminate asexual parasites. Two biological replicates were performed. Control samples of uninfected RBCs, were cultured in parallel, in the same conditions. Asexual stage parasitemia, gametocytaemia, and gametocyte morphology were determined by microscopic inspection of Giemsa-stained thin smears.

Culture medium, from infected and non-infected cultures, were collected and centrifuged at 800 g for 10 min. Supernatants were stored until analysis at −80 °C.

Human blood from adult healthy volunteer donors was used to maintain blood stage cultures of *P. falciparum*. The blood and protocol were approved for use by the Blood Transfusion Unit at the Sapienza University of Rome, Italy. All methods were performed in accordance with relevant guidelines and regulations on suitability assessment of blood donors and blood components (Ministry of Health-Decree of 3 march 2005, Official Gazette no 85, 13-4-2005). Donors provided informed written consent for use of their blood for research purposes.

### VOCs analysis by PTR-ToF-MS

The head-space composition of supernatant samples was analyzed by direct injection in a commercial PTR-ToF-MS 8000 apparatus (Ionicon Analytik GmbH, Innsbruck, Austria) after passing a Gas Sensor Array (see next paragraph) both directly for 30 s and after, by fastGC add-on coupled to PTR-ToF-MS. Samples were handled by a multipurpose GC automatic sampler (Autosampler, Gerstel GmbH, Mulheim am Ruhr, Germany)^28^. The instrumental conditions in the drift tube were as follows: drift voltage 628 V, drift temperature 110 °C, drift pressure 2.80 mbar, producing an E/N value of 130 Td (1 Td = 10^−17^ Vcm^2^), where E is the electric field strength and N is the gas number density. In order to increase the sensitivity an ion funnel was operated at the end of the drift tube^29^. The sampling time per channel of ToF acquisition was 0.1 ns, amounting to 350,000 channels for a mass spectrum ranging up to m/z = 400. Every single spectrum is the sum of 28600 acquisitions lasting 35 μs each, resulting in a time resolution of  1 s. A certified mixture of 15 volatile compounds spanning the mass range from 34 (protonated methanol) to 148 (protonated dichlorobenzene) produced by a gas calibration unity (GCU, Ionicon Analytik, Innsbruck, Austria) allows a check of the concentration estimated by PTR-MS.

VOC measurements were performed in triplicate on 3 mL of sample collected in 20 mL vials. Samples were stored at 4 °C and before analysis were incubated for 30 min at 37 °C and measured for 30 sec in a direct mode (i.e. without passing through the fastGC column), then the volatile mixture was diverted towards the fastGC loop for further 30 sec. Between measurements, 5 min interval was kept to complete the fastGC chromatographic run and avoid memory effects.

The polar capillary column [MXT®-WAX (Siltek® - treated stainless steel), 6 m] of a fastGC add-on was maintained under pure N_2_ with a constant flow rate of 3 sccm. The chromatographic measurement was registered for 155 s with a constant slope thermal ramp from 40 °C to 220 °C.

### Sensor array

The gas sensor array was an ensemble of twelve quartz microbalances (QMB). In these sensors, a mass change (Δm) on the quartz surface results in frequency changes (Δf) of the electrical output signal of an oscillator circuit at which each sensor is connected. In the low-perturbation regime, Δm and Δf are linearly proportional^30^. QMBs had a fundamental frequency of 20 MHz, corresponding to a mass resolution of the order of a few nanograms.

The sensor system used in these experiments was the last version of a series of instruments designed since 1996 at the University of Rome Tor Vergata. The gas sensors are complemented by temperature and relative humidity sensors. Each QMB is connected to an oscillator circuit, the frequency of the oscillator’s outputs are measured taking advantage of a temperature compensated reference quartz that allows for a frequency resolution of 0.1 Hz. Electronics is implemented in a FPGA. Gaseous samples delivery is controlled by a miniature diaphragm pump (0–200 sccm). The instrument is connected and powered via a single USB connection. Functions and data acquisition are controlled with an in-house software running in Matlab.

The functional sensing materials are the same porphyrins and corroles previously used to measure the VOCs released by malaria infected mice^7^.

### Data processing and statistical analysis

Data processing of PTR-ToF-MS spectra included dead time correction, external calibration and peak extraction steps performed according to a procedure described elsewhere^31^. The baseline of the mass spectra was removed after averaging the whole measurement and peak detection and peak area extraction was performed by using a modified Gaussian to fit the data^32^. To determine the concentrations of volatile compounds in ppbv (part per billion by volume) the formulas described by Lindinger *et al*.^33^ were used assuming a constant reaction rate coefficient (kR = 2 × 10^−9^ cm^3^/s) for H_3_O+ as primary ion.

The statistical significance of PTR-MS peaks, sensors signals, and PCA scores have been evaluated with the non parametric Kruskal-Wallis rank sum test followed by Bonferroni correction in case of multiple comparisons.

The absolute abundances of PTR-MS peaks and the sensor responses were arranged in matrices and then analysed with multivariate data analysis. Principal component analysis (PCA) was calculated on intact data for PTR-MS and on autoscaled data matrices in case of sensors signals^7^. In autoscaling each variable of the matrix was normalized to null mean and unitary variance. Variance analysis and multivariate analysis were performed in Matlab R2017a, PCA was calculated with the Statistics and Machine Learning toolboxes of Matlab.

## Supplementary information

SUPPLEMENTARY INFORMATION
