# Supplementary material for: Simultaneous Proton Transfer Reaction-Mass Spectrometry and electronic nose study of the volatile compounds released by Plasmodium falciparum infected red blood cells in vitro
Source: Sci Rep. 2019 Aug 26;9:12360. doi: 10.1038/s41598-019-48732-x (PMC6710240; doi:10.1038/s41598-019-48732-x)
Supplement: Supplementary file 1 — SUPPLEMENTARY INFORMATION [file 41598_2019_48732_MOESM1_ESM.docx]

**Simultaneous Proton Transfer Reaction-mass spectrometry and electronic nose study of the volatile compounds released by *Plasmodium falciparum* infected red blood cells *in vitro*.**

*Rosamaria Capuano^1^, Iuliia Khomenko^2^, Felicia Grasso^3^, Valeria Messina^3^, Anna Olivieri^3^, Luca Cappellin^4^, Roberto Paolesse^5^, Alexandro Catini^1^, Marta Ponzi^3§^, Franco Biasioli^2§^, Corrado Di Natale^1§^*

1 Department of Electronic Engineering, University of Rome Tor Vergata, Via del Politecnico 1, 00133 Roma, Italy

2 Dept. Food Quality and Nutrition, Fondazione E. Mach. Via E. Mach 1, 38010 S. Michele all’Adige (TN), Italy

3 Department of Infectious Diseases, Istituto Superiore di Sanità, Viale Regina Elena 299, 00161 Roma, Italy

4 Department of Chemical Sciences, University of Padua, Via F. Marzolo 1, 35131 Padova, Italy

5 Department of Chemical Science and Technology, University of Rome Tor Vergata, Via della Ricerca Scientifica, 00133 Rome, Italy

§ correspondence to: dinatale@uniroma2.it, marta.ponzi@iss.it, franco.biasioli@fmach.it

**SUPPLEMENTARY INFORMATION**

Figure S1: *concentration of m=84.0889 in all samples. This mass corresponds to the isotopologue of mass m=83.0856. identified as Hexanal.*

Figure S2: Statistical distribution of each mass detected by PTR-MS in the first (A) and second (B) parts of the experiment. The discriminant p<0.01 is indicated in each plot.

Figure S3: explained variance of the principal components of PTR-MS data related to intact red blood cells and asexuated *Plasmodium*.

Figure S4: statistical distribution of the first six principal components of the PTR-MS spectra of intact red blood cells and asexuated *Plasmodium*. The header of each plot shows the correspondent p-value related to at least the separation of two groups.

Figure S5: explained variance of the principal components of PTR-MS data related to intact red blood cells and *Plasmodium* gametocytes.

Figure S6: statistical distribution of the first six principal components of the PTR-MS spectra of intact red blood cells and *Plasmodium* gametocytes. The header of each plot shows the correspondent p-value related to at least the separation of two groups.

Figure S7: statistical distribution of the first four principal components of the electronic nose data related to intact red blood cells and asexuated *Plasmodium*. The header of each plot shows the correspondent p-value related to the separation of at least two groups.

Figure S8: statistical distribution of the first four principal components of the electronic nose data relate intact red blood cells and gametocytes. The header of each plot shows the correspondent p-value related to the separation of at least two groups.
